# Supplementary material for: Pisces: A multi-modal data augmentation approach for drug combination synergy prediction
Source: Cell Genom. 2025 Jun 3;5(7):100892. doi: 10.1016/j.xgen.2025.100892 (PMC12278649; doi:10.1016/j.xgen.2025.100892)
Supplement: Document S1. Figures S1–S19 and Tables S1–S4 [file mmc1.pdf]

Cell Genomics, Volume *5*

Supplemental information

Pisces: A multi-modal data augmentation approach for drug combination synergy prediction

Hanwen Xu, Jiacheng Lin, Addie Woicik, Zixuan Liu, Jianzhu Ma, Sheng Zhang, Hoifung Poon, Liewei Wang, and Sheng Wang

Supplementary Infomation

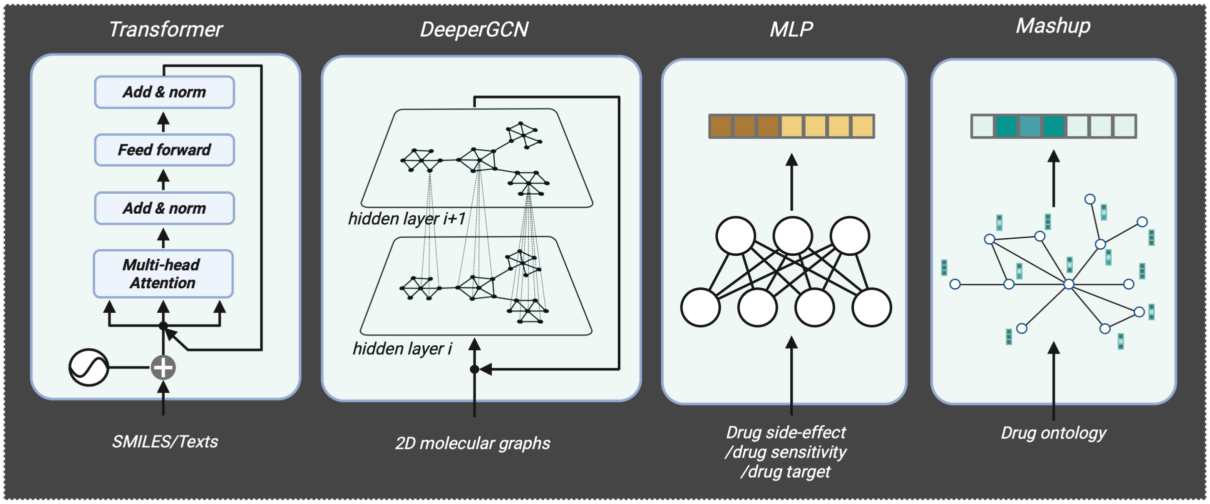

Supplementary Fig. 1 Model architectures of encoders for different modalities, related to Figure 1. We used Transformer architectures for SMILES and textual descriptions, DeeperGCN for molecular graphs (including encoders aligned with 3D geometric views). We used MLP layers for drug sensitivity, drug side effects and drug targets. We finally used Mashup to extract the drug ontology structures.

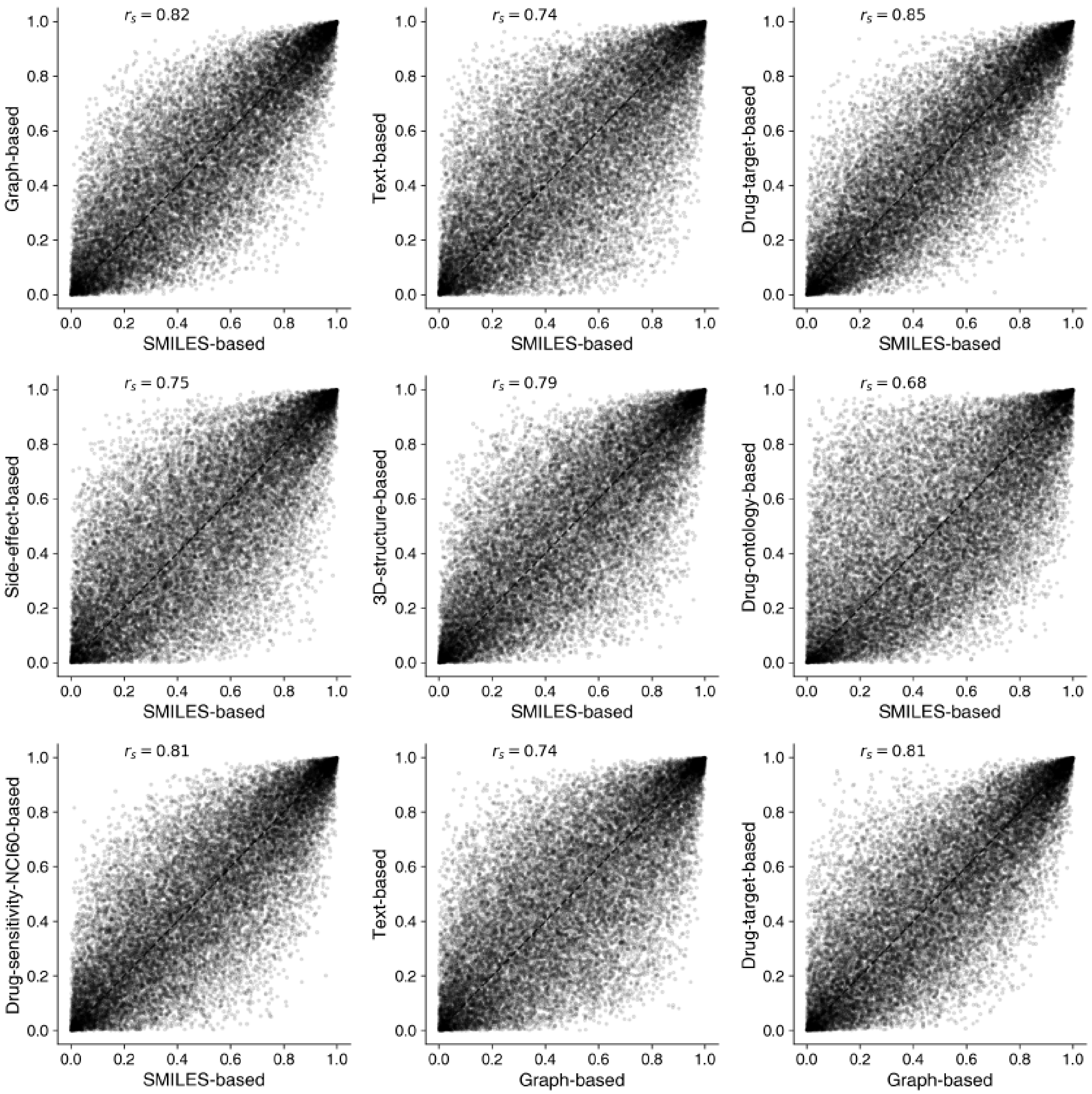

Supplementary Fig. 2 The agreement among predictions of different modalities, related to Figure 2. Scatter plot comparing the prediction scores of using different modalities. We separately performed normalized ranking for predictions based on each modality. The correlations between modalities were measured using Spearman correlation.

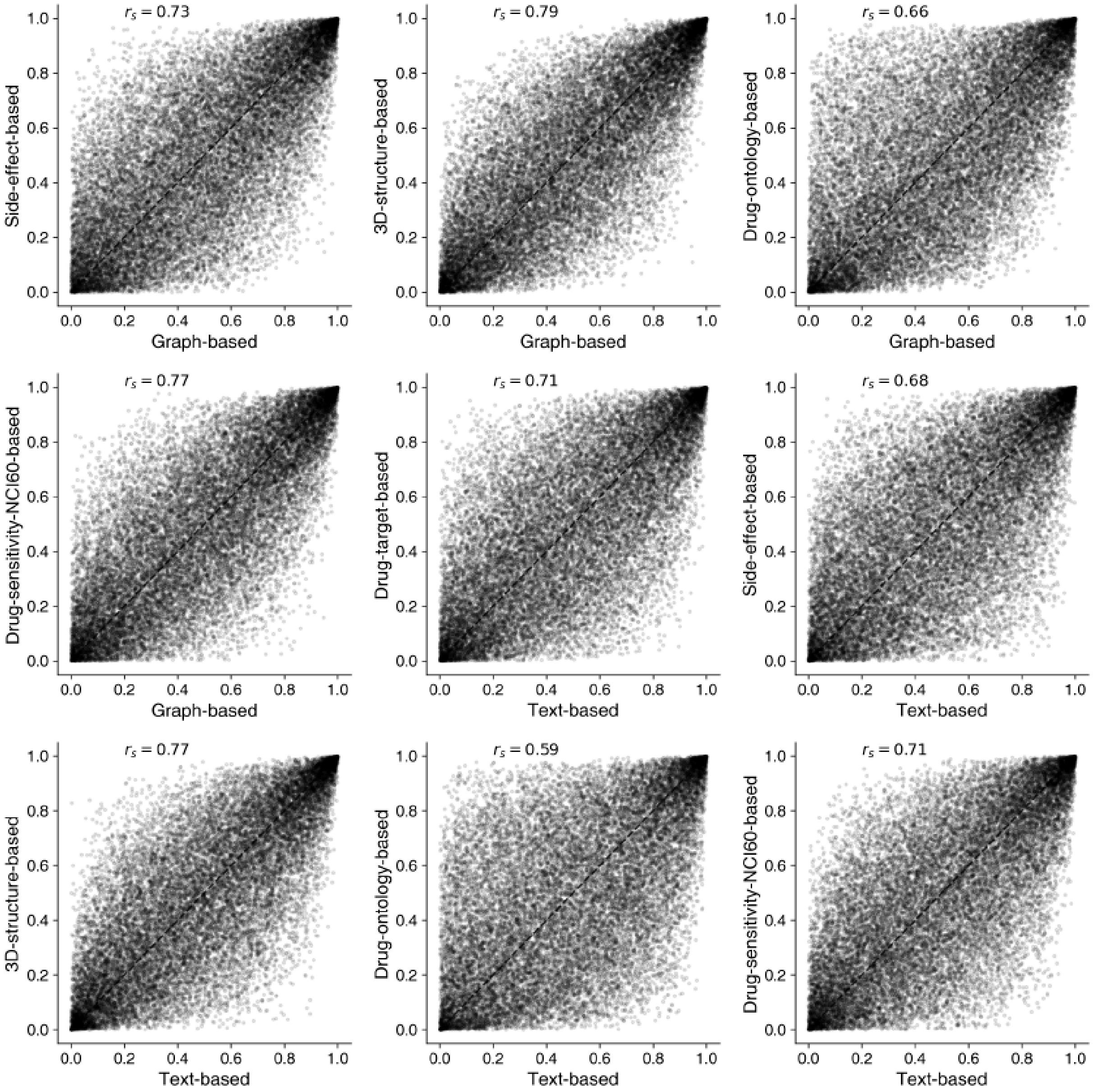

Supplementary Fig. 3 The agreement among predictions of different modalities, related to Figure 2. Scatter plot comparing the prediction scores of using different modalities. We separately performed normalized ranking for predictions based on each modality. The correlations between modalities were measured using Spearman correlation.

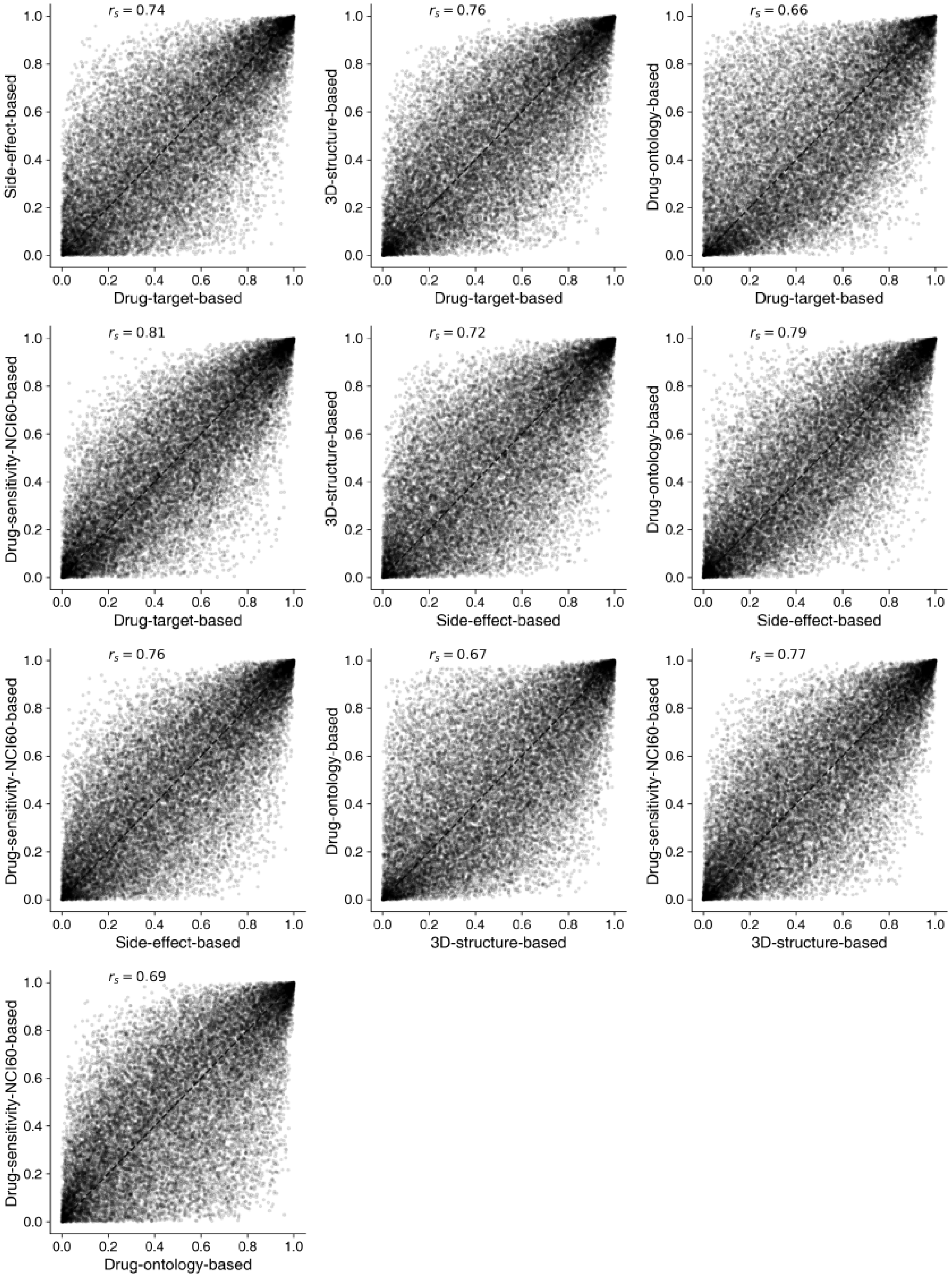

Supplementary Fig. 4 The agreement among predictions of different modalities, related to Figure 2. Scatter plot comparing the prediction scores of using different modalities. We separately performed normalized ranking for predictions based on each modality. The correlations between modalities were measured using Spearman correlation.

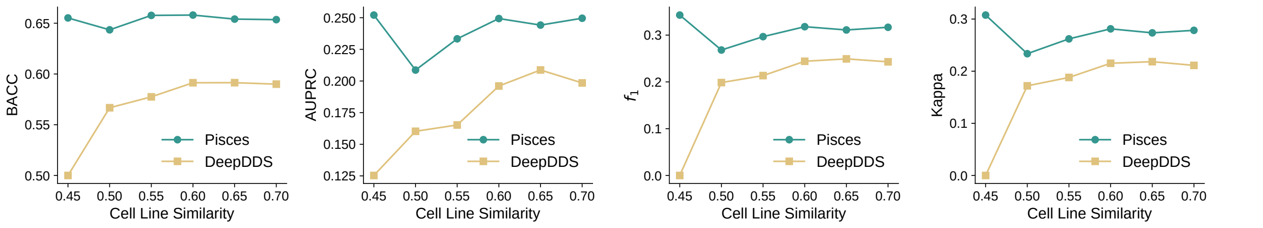

Supplementary Fig. 5 Comparison in the split by cell line setting with varying similarities between training and testing cell lines, related to Figure 3. Plots showing how the performance changes by controlling the similarities between training and testing cell lines. The similarity score is calculated as the jaccard similarity between the overexpressed gene sets of two cell lines. The overexpressed gene set is determined in the same way as we determined that for the cell-line based prediction task in Pisces.

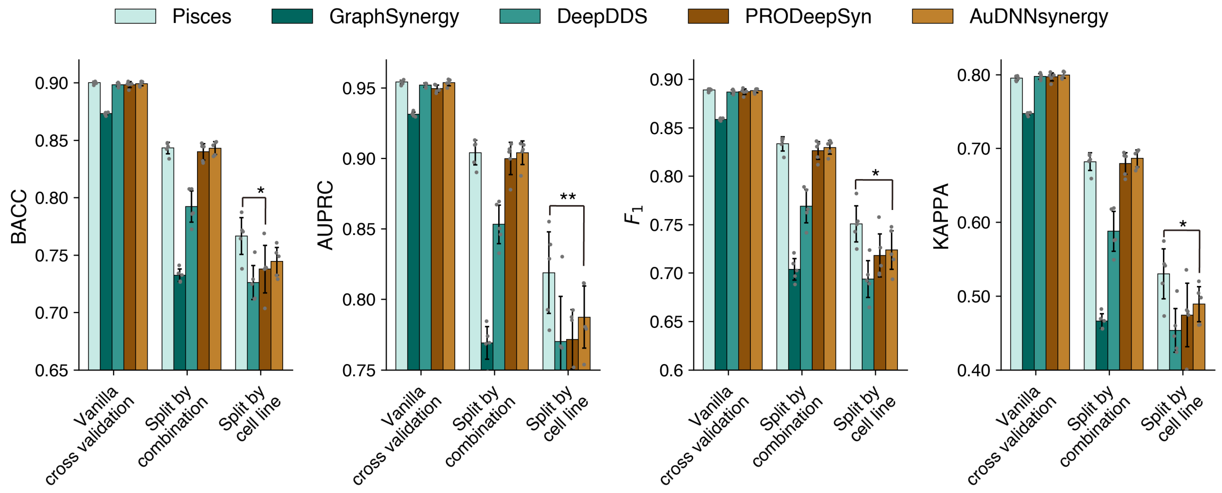

Supplementary Fig. 6 Comparison on DrugCombDB dataset, related to Figure 3. Bar plots comparing the drug synergy prediction on DrugCombDB under three data split settings (x-axis) using BACC, AUPRC, F1, Cohen's Kappa. Split by combination means all test combinations have never been seen in the training data. Split by cell line means all test cell lines have never been seen in the training data. The * indicates that Pisces outperforms the next-best-performing model in the metric, with significance levels of t-test p-value $< 5e\text{-}2$ for *, t-test p-value $< 1e\text{-}2$ for **, and t-test p-value $< 1e\text{-}3$ for ***.

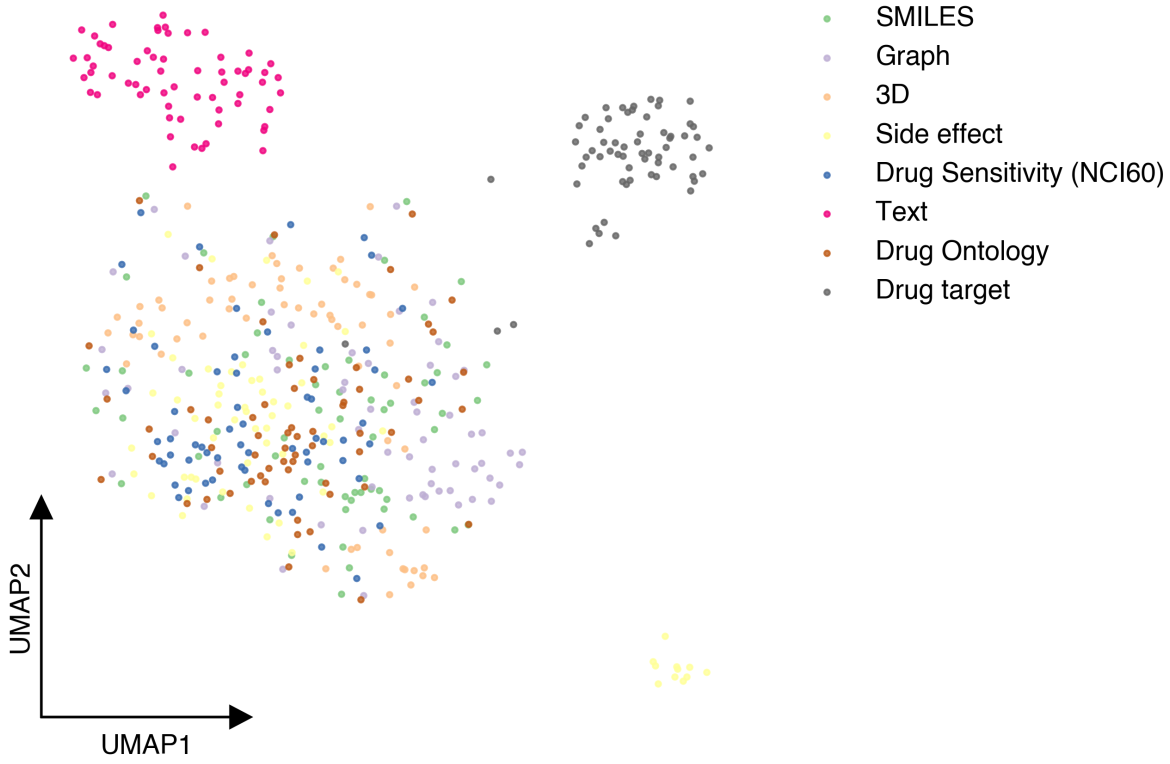

Supplementary Fig. 7 Feature embedding space visualization, related to Figure 3. UMAP visualization showing the distribution of features from 8 modalities. Each dot represents one feature of a modality from a specific drug. Each color represents a specific modality.

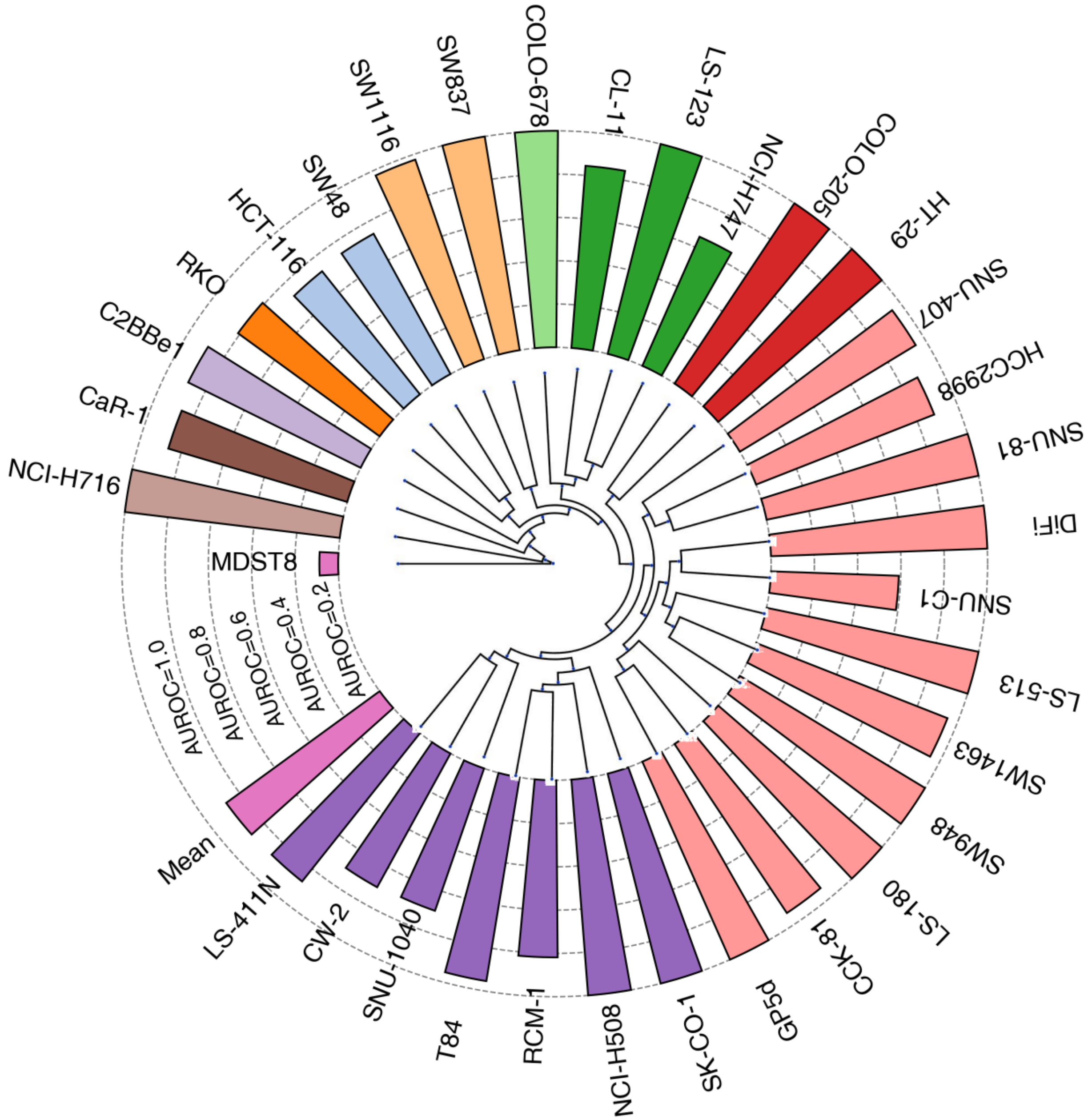

Supplementary Fig. 8 Three-drug combination prediction in terms of AUROC, related to Figure 3. Circular bar plots showing Pisces' prediction performance in terms of AUROC on three-drug combination prediction when trained only on two-drug and single-drug data. AUROC are only shown stratified by cell line features. The circular dendrogram shows the hierarchical clustering of cell lines using gene expression levels. The branch height represents the distances between two cell line clusters.

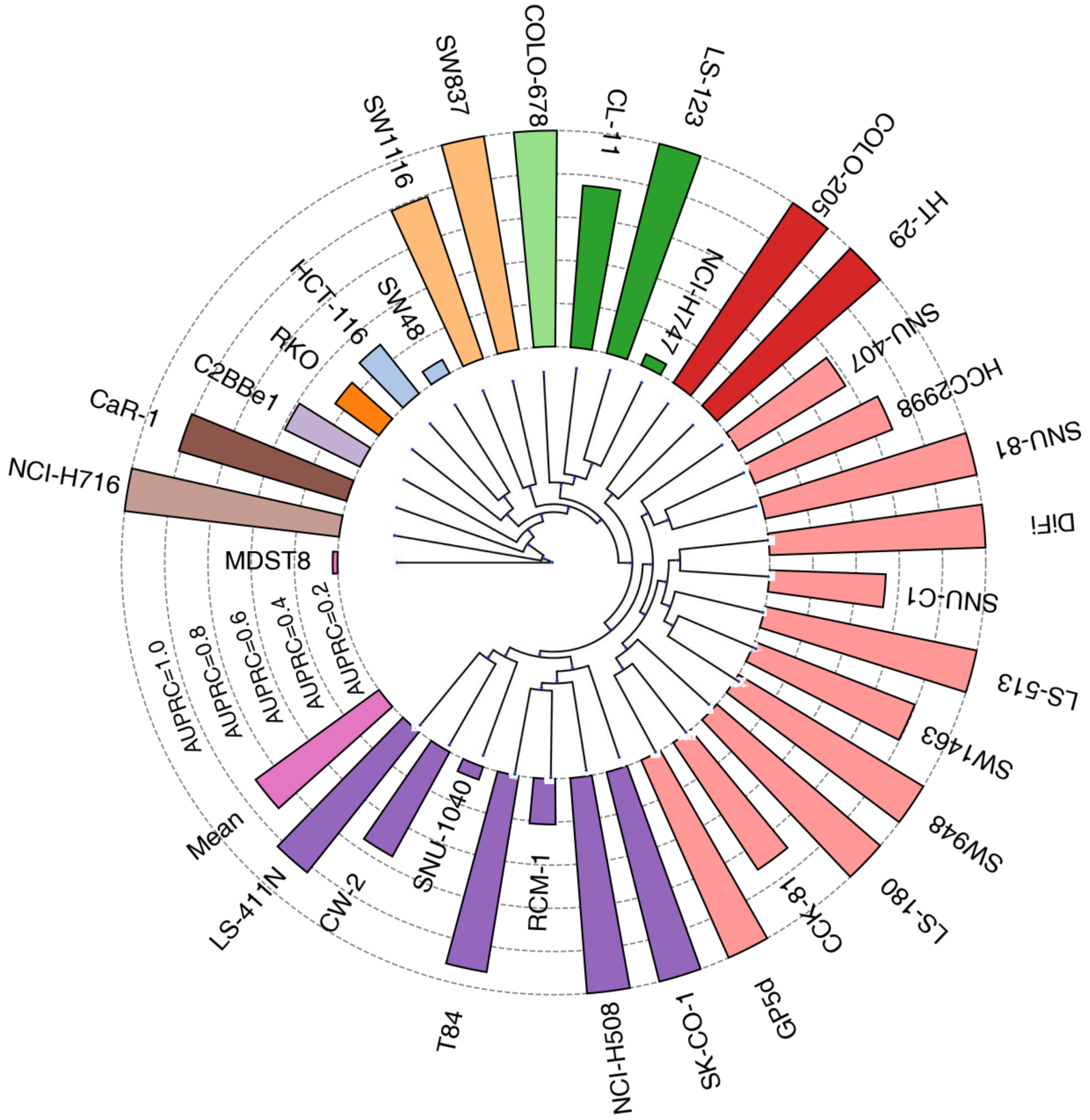

Supplementary Fig. 9 Three-drug combination prediction in terms of AUPRC, related to Figure 3. Circular bar plots showing Pisces' prediction performance in terms of AUPRC on three-drug combination prediction when trained only on two-drug and single-drug data. AUPRC and AUPRC are only shown stratified by cell line features. The circular dendrogram shows the hierarchical clustering of cell lines using gene expression levels. The branch height represents the distances between two cell line clusters.

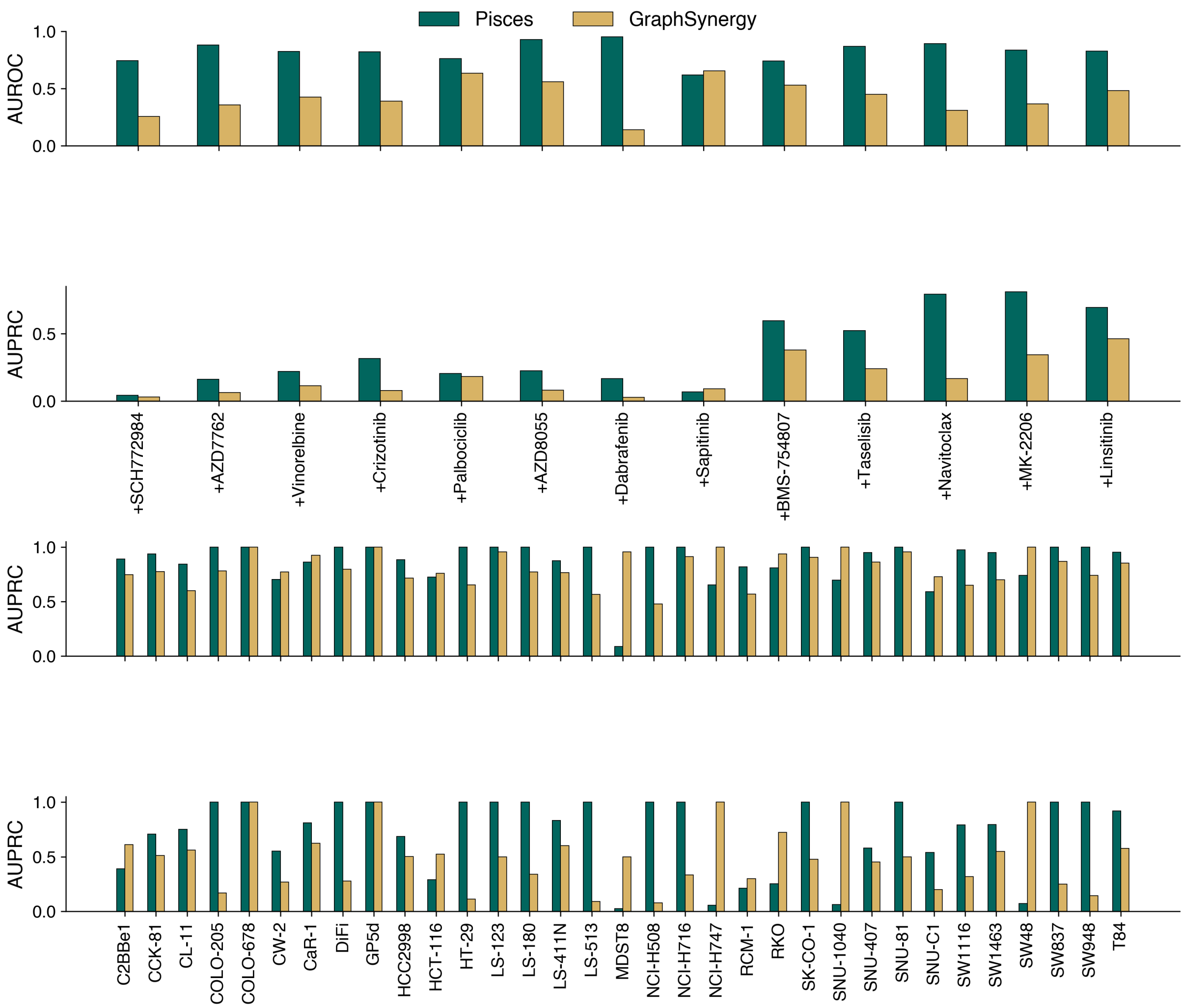

Supplementary Fig. 10 Comparison on three-drug combination predictions, related to Figure 3. Bar plot showing the AUROC and AUPRC of Pisces and GraphSynergy on ranking cell lines for a three-drug combination or ranking three-drug combinations for a cell line.

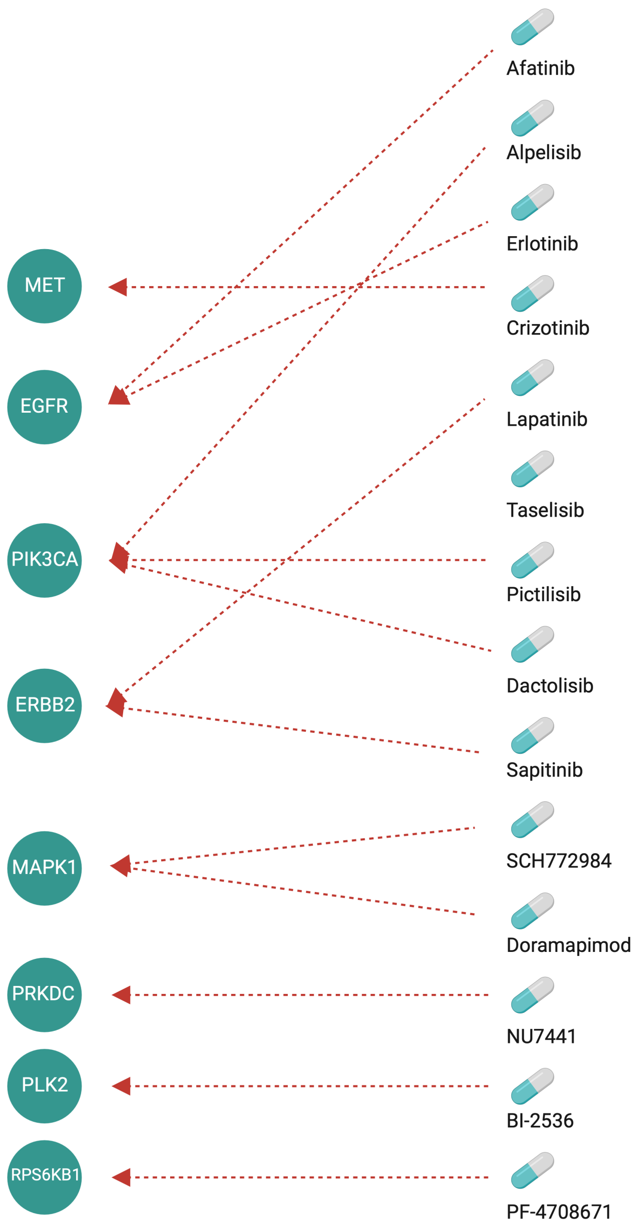

Supplementary Fig. 11 Bipartite graph of drugs and target genes, related to Figure 3. Bipartite graph showing 14 cancer drugs and 8 genes that can be inhibited by one of these drugs. Each edge links one drug and the gene that can be inhibited by this drug.

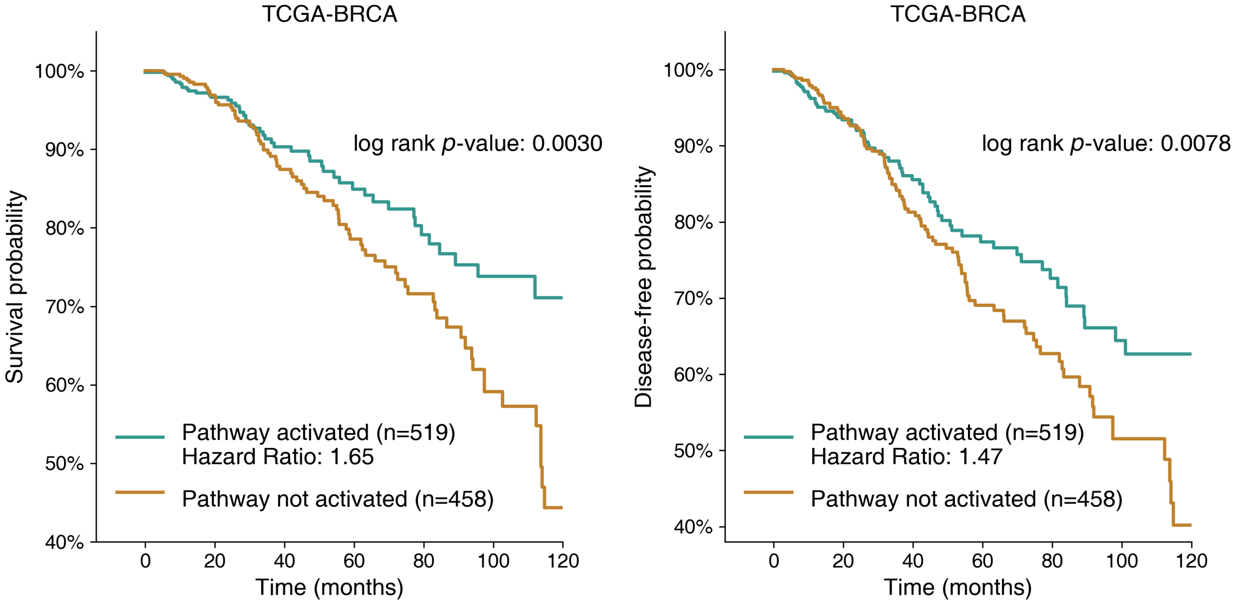

Supplementary Fig. 12 Survival analysis on patients from TCGA dataset, related to Figure 3. Survival plots showing the significantly different overall survival (left) and disease-free survival (right) between two groups of TCGA-BRCA patients. These two groups were clustered based on whether the BRCA drug-sensitive pathway was activated using the gene expression.

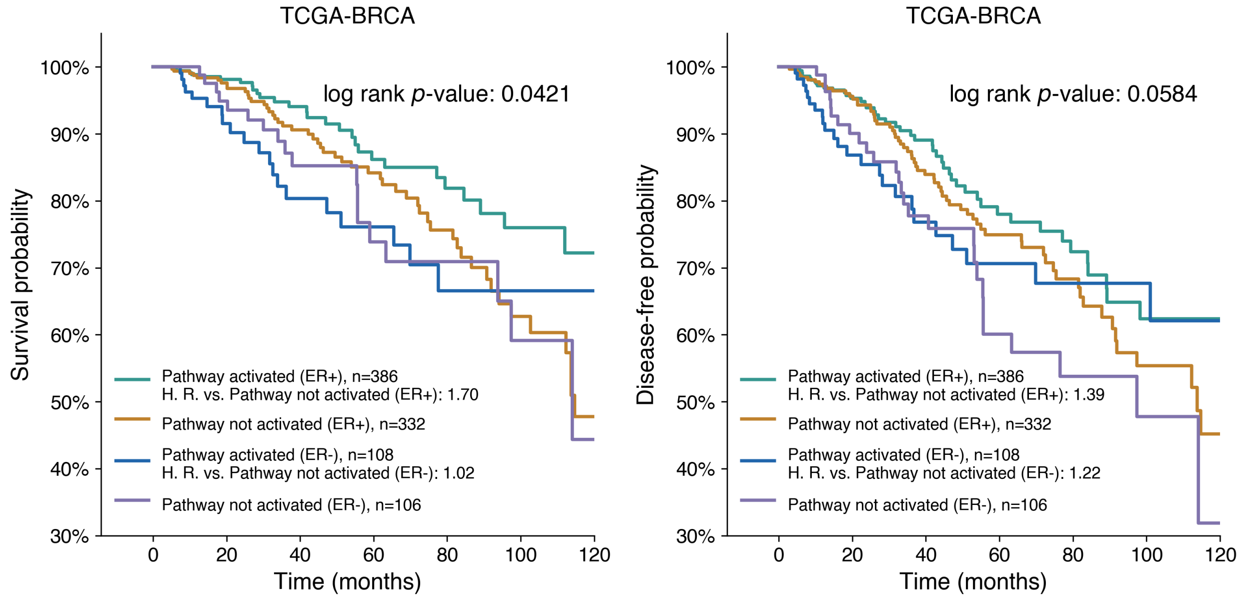

Supplementary Fig. 13 Survival analysis on patients from TCGA dataset using the gene expression and ER status after excluding BRCA cell lines from training data, related to Figure 3. Survival plots showing the significantly different overall survival and disease-free survival among four groups of TCGA-BRCA patients. The BRCA cancer cell lines have been excluded from the Pisces training data. These four groups are classified using the gene expression in the BRCA drug-sensitive pathway and ER status. .

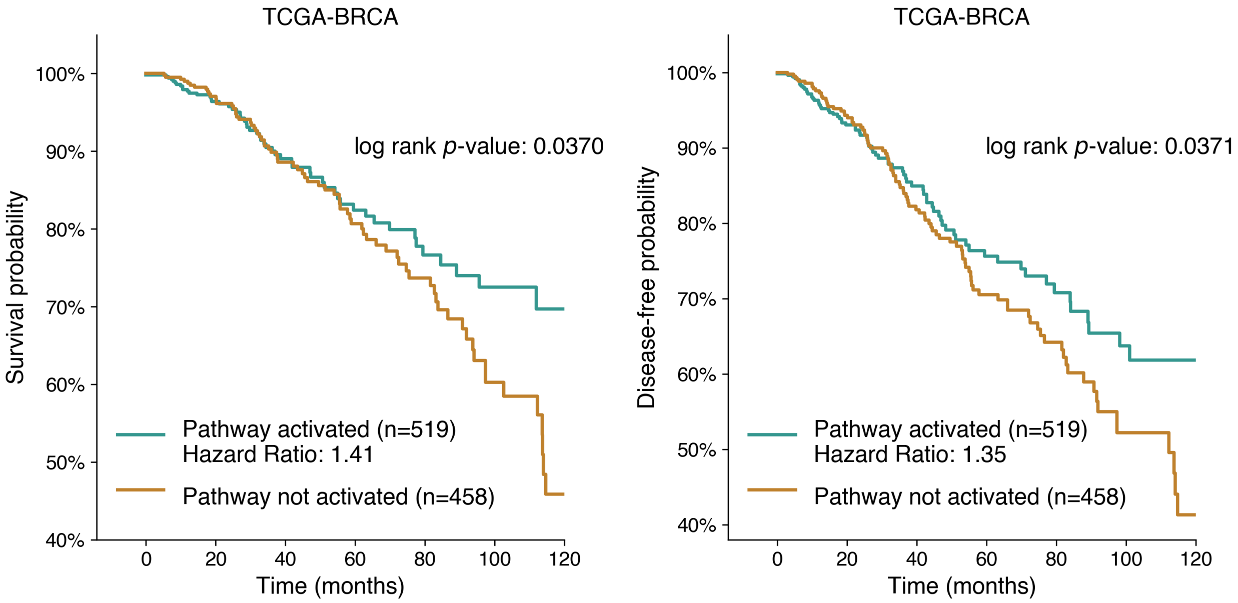

Supplementary Fig. 14 Survival analysis on patients from TCGA dataset after excluding BRCA cell lines from training data, related to Figure 3. Survival plots showing the significantly different overall survival (left) and disease-free survival (right) between two groups of TCGA-BRCA patients. The BRCA cancer cell lines have been excluded from the Pisces training data.These two groups were clustered based on whether the BRCA drug-sensitive pathway was activated using the gene expression.

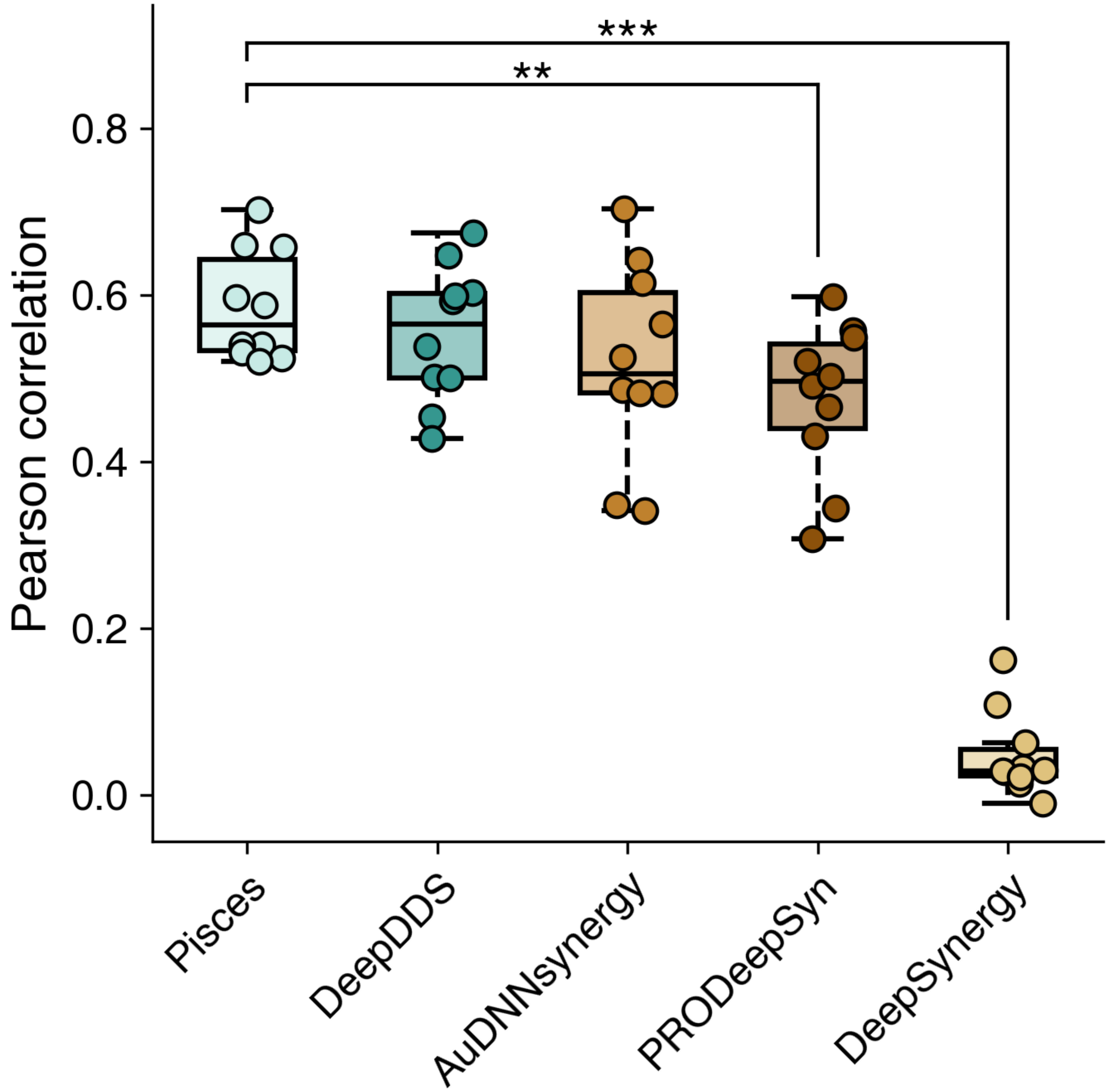

Supplementary Fig. 15 Tumor change prediction in terms of Pearson correlation, related to Figure 4. Box plot comparing the drug synergy prediction on xenografts using Pearson correlation. The * indicates that Pisces outperforms the next-best-performing model in the metric, with significance levels of t-test p-value $< 5 \times 10^{-2}$ for *, t-test p-value $< 1 \times 10^{-2}$ for **, and t-test p-value $< 5 \times 10^{-3}$ for ***. All t-tests are one-sided.

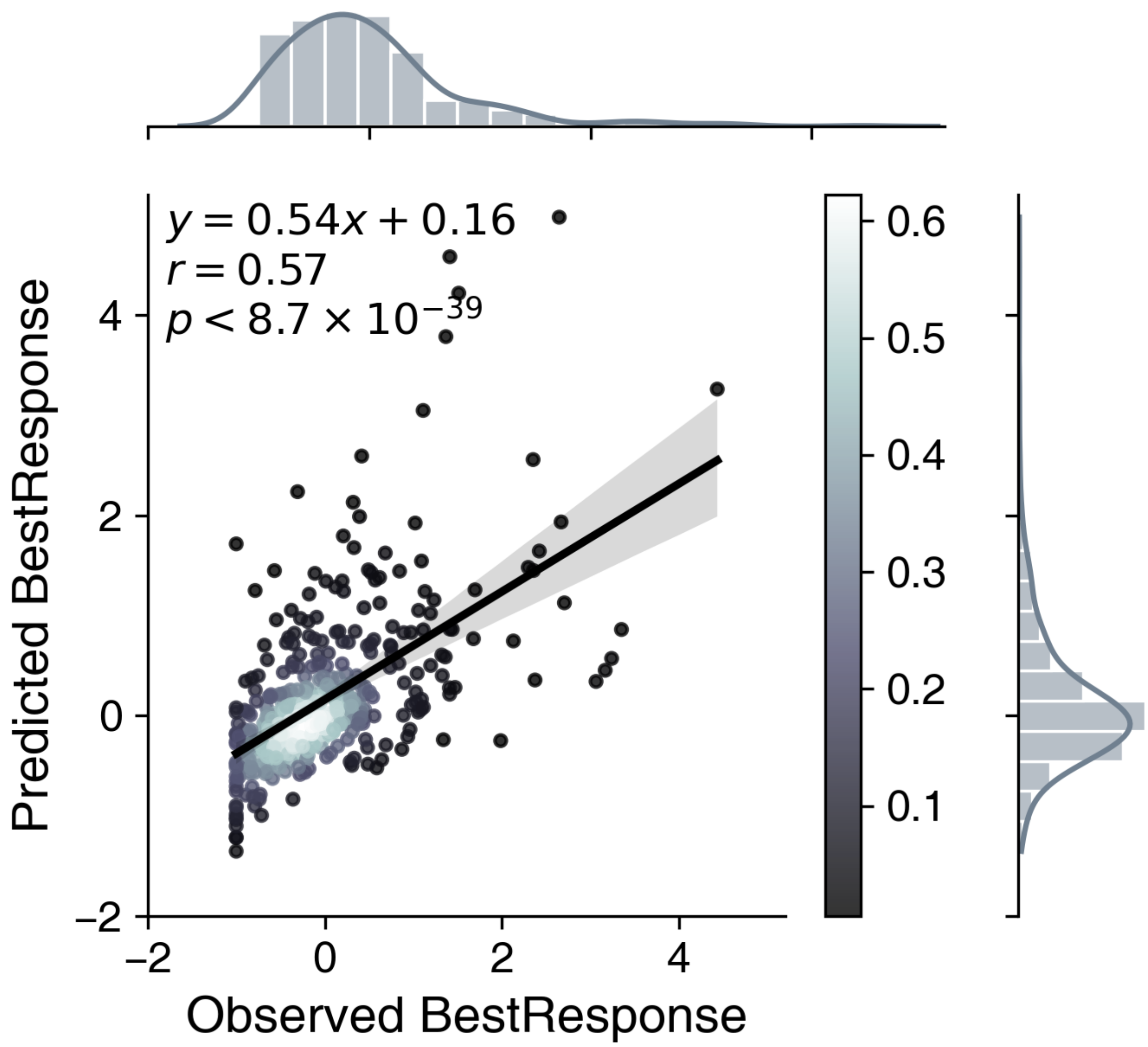

Supplementary Fig. 16 Scatter plot of tumor change prediction, related to Figure 4. Scatter plots comparing the predicted and observed minimum %tumor changes after 10 days at holdout triplets. Bar plots show the frequency.

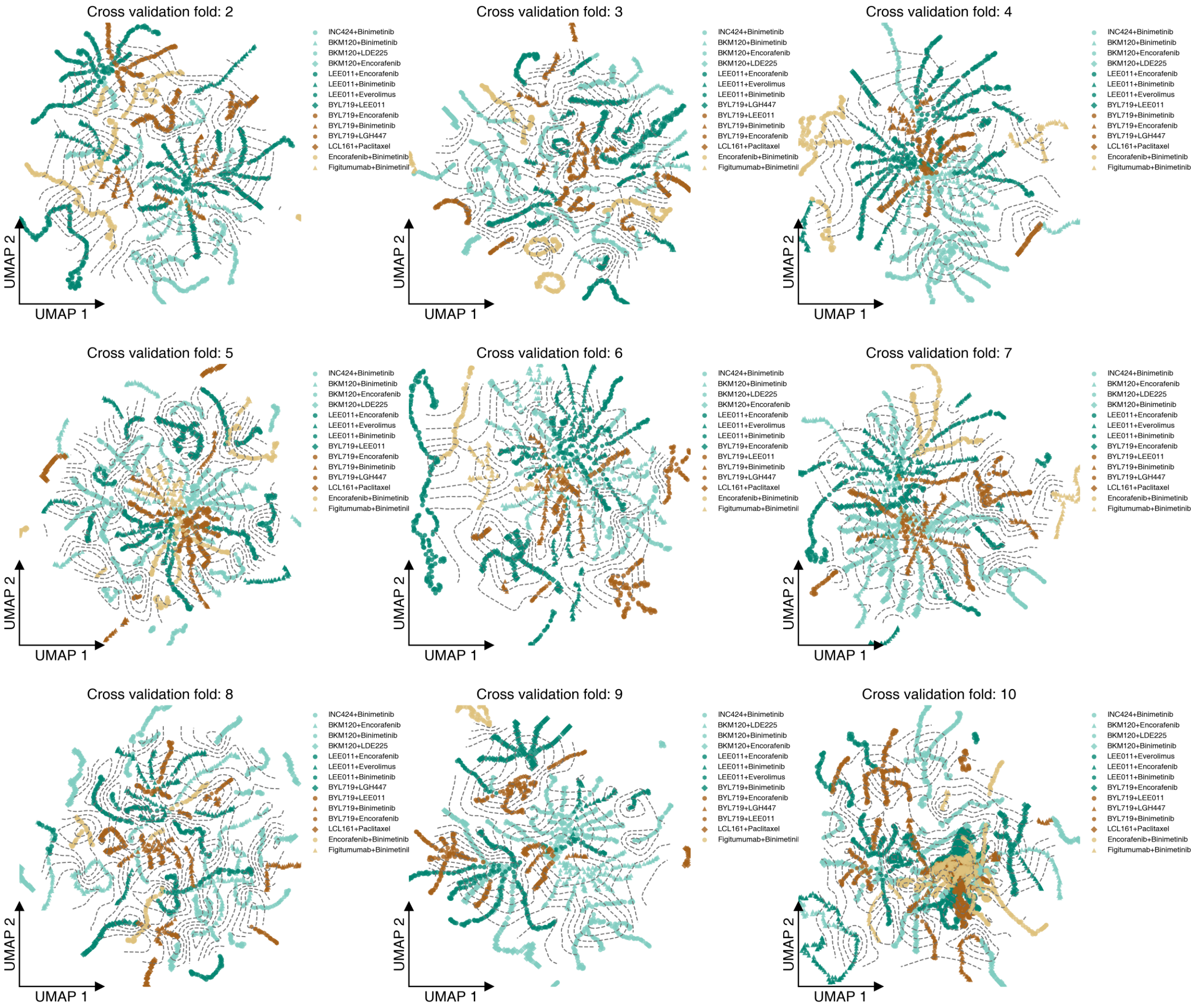

Supplementary Fig. 17 UMAP plot of test triplets at different time points, related to Figure 4. UMAP plot showing the embedding of test triplets at different time points from 9 other folds in the cross validation. Each triplet is a pair of drugs and a xenograft model. Nodes are colored and marked by the drug pair.

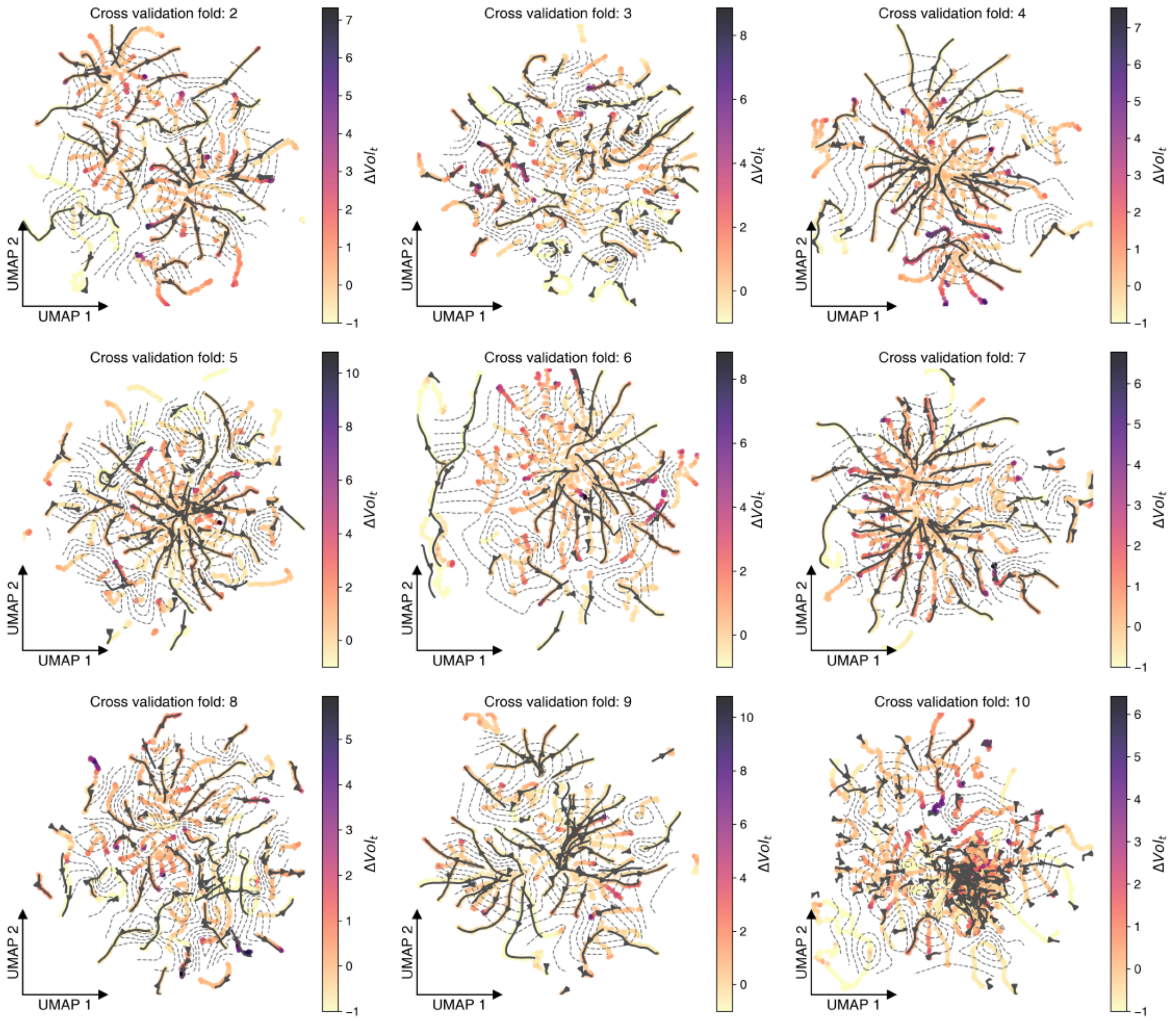

Supplementary Fig. 18 UMAP plot of one drug pair on different xenografts, related to Figure 4. UMAP plot showing the embedding of test triplets at different time points from 9 other folds in the cross validation. Each triplet is a pair of drugs and a xenograft model. The nodes are colored and marked by the tumor volume change. The contours connect tumors that have the same time point. The arrows are from early time points to later time points.

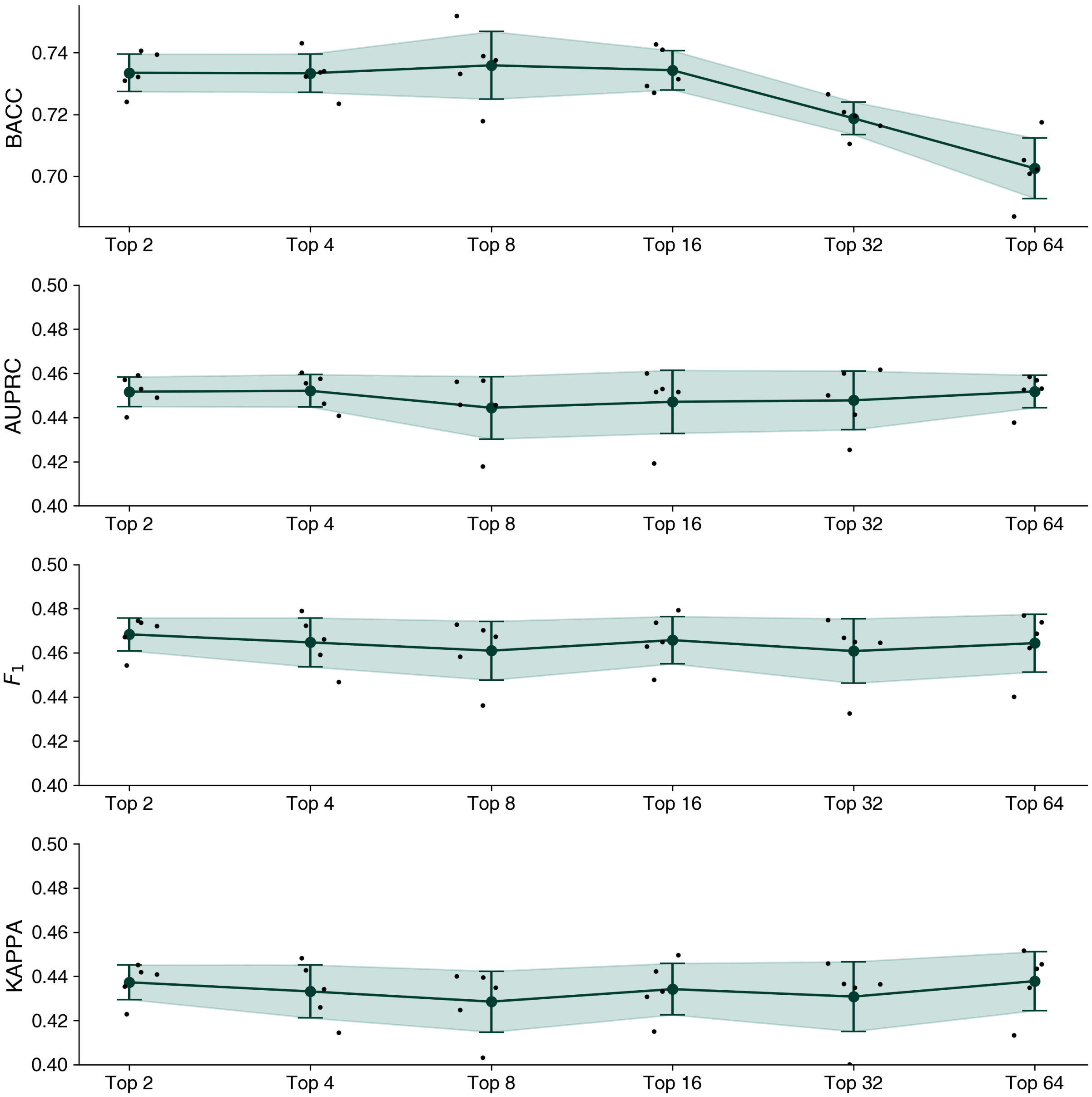

Supplementary Fig. 19 Ablation studies on various top k, related to STAR Methods. UMAP plot showing the performance changes by aggregating various top k predictions. The x axis show the aggregation of top 2, 4, 8, 16, 32 and all 64 predictions. We investigated on the GDSC-Combo dataset in the vanilla cross validation setting. Performances were measured in terms of BACC, AUPRC, F_1 and KAPPA. Error bar represents the standard deviation across 5 fold cross validation. Each white dot represents on one fold.

Drug A Class	Drug B Class	Drug-drug interactions	p-value	Number of interactions
phenols	homopolycyclic compound	Increase the anticholinergic activities	5.63×10^{-9}	43
zwitterion	organofluorine compound	Increase the risk or severity of QTc prolongation	2.40×10^{-4}	31
organochlorine compound	phenols	Decrease the vasoconstricting activities	1.12×10^{-10}	26
zwitterion	organochlorine compound	Decrease the diuretic activities	1.30×10^{-7},26	
organochlorine compound	homopolycyclic compound	Increase the constipating activities	4.80×10^{-7}	22
organic fundamental parent	homopolycyclic compound	Decrease the sedative activities	1.54×10^{-9},21	
organochlorine compound	hydroxy steroid	Increase the thrombogenic activities	3.59×10^{-10}	18
organofluorine compound	phenols	Decrease the vasoconstricting activities	3.13×10^{-11}	17
phenols	organic fundamental parent	Increase the anticholinergic activities	6.03×10^{-7}	17
homopolycyclic compound	phenols	Increase the thrombogenic activities	8.91×10^{-5}	16
organofluorine compound	homopolycyclic compound	Increase the anticholinergic activities	2.64×10^{-5}	14
organic fundamental parent	organofluorine compound	Increase the thrombogenic activities	2.93×10^{-4}	14
aromatic amine	homopolycyclic compound	Decrease the sedative activities	2.40×10^{-7}	12
sulfonic acid derivative	organochlorine compound	Decrease the diuretic activities	4.21×10^{-3}	9
sulfonic acid derivative	hydroxy steroid	Increase the thrombogenic activities	1.52×10^{-6}	9
aromatic amine	hydroxy steroid	Increase the thrombogenic activities	1.05×10^{-5}	6
olefinic compound	hydroxy steroid	Increase the thrombogenic activities	4.61×10^{-3}	5
organofluorine compound	organic fundamental parent	Decrease the vasoconstricting activities	5.83×10^{-5}	4
olefinic compound	phenols	Increase the anticholinergic activities	2.99×10^{-4}	3
homopolycyclic compound	hydroxy steroid	Increase the thrombogenic activities	8.17×10^{-3}	3
sulfonic acid derivative	organic fundamental parent	Increase the risk or severity of QTc prolongation	1.75×10^{-4}	3

Supplementary Table 1 Table showing drug-drug interactions significantly occurred between two drug classes, related to Figure 5. The first three columns indicate two drug classes and the associated interaction type . The p-value represented the Fisher's exact test results. The number of interactions means how many interactions found between drugs from these two classes.

	BACC	AUPRC	F1	KAPPA
Pisces	0.7359	0.4444	0.4609	0.4285
w/o consistency loss	0.7024	0.4341	0.4515	0.4232
w/o InfoNCE loss	0.6978	0.4353	0.4498	0.4220
top k = 1	0.7044	0.4473	0.4624	0.4352

Supplementary Table 2 Table showing ablation study results on GDSC dataset transductive setting, related to Figure 3. All experiments are run across five folds.

	BACC	AUPRC	F1	KAPPA
Pisces	0.7359	0.4444	0.4609	0.4285
Concat all 8 features	0.7031	0.4398	0.4519	0.4235
SMILES, Graph, 3D, Side effects, Drug Sensitivity	0.7045	0.4349	0.4543	0.4259
SMILES, Graph, Text, Drug Ontology, Drug target	0.7022	0.4346	0.4551	0.4272

Supplementary Table 3 Table showing study results for modality combination on GDSC dataset transductive setting, related to Figure 3. All experiments are run across five folds.

	BACC	AUPRC	F1	KAPPA
Pisces	0.7359	0.4444	0.46096	0.4285
max position 128	0.6444	0.4277	0.3945	0.3719
memory 16	0.7109	0.4509	0.4609	0.4325
learning rate 1e-5	0.6934	0.4616	0.4595	0.4340

Supplementary Table 4 Table showing sensitive analysis results on GDSC dataset transductive setting, related to Figure 3. All experiments are run across five folds. The max position, memory, and learning rate for Pisces are 256, 32, and 5e-5.
